# Socially induced negative affective knowledge modulates early face perception but not gaze cueing of attention

**DOI:** 10.1111/psyp.13876

**Published:** 2021-06-10

**Authors:** Magdalena Matyjek, Bartłomiej Kroczek, Magdalena Senderecka

**Affiliations:** ^1^ Institute of Philosophy Jagiellonian University Kraków Poland; ^2^ Faculty of Mathematics and Computer Science Jagiellonian University Kraków Poland; ^3^ Present address: Berlin School of Mind and Brain, Department of Psychology Humboldt‐Universität zu Berlin Berlin Germany; ^4^ Present address: Department of Psychology Humboldt‐Universität zu Berlin Berlin Germany; ^5^ Present address: Institute of Psychology Jagiellonian University Kraków Poland

**Keywords:** affect induction, affective knowledge, EEG, ERP, gaze‐cueing tasks, P1, social interaction

## Abstract

Prior affective and social knowledge about other individuals has been shown to modulate perception of their faces and gaze‐related attentional processes. However, it remains unclear whether emotionally charged knowledge acquired through interactive social learning also modulates face processing and attentional control. Thus, the aim of this study was to test whether affective knowledge induced through social interactions in a naturalistic exchange game can influence early stages of face processing and attentional shifts in a subsequent gaze‐cueing task. As indicated by self‐reported ratings, the game was successful in inducing valenced affective knowledge towards positive and negative players. In the subsequent task, in which the locations of future targets were cued by the gaze of the game players, we observed enhanced early neural activity (larger amplitude of the P1 component) in response to a photograph of the negative player. This indicates that negative affective knowledge about an individual indeed modulates very early stages of the processing of this individual's face. Our study contributes to the existing literature by providing further evidence for the saliency of interactive social exchange paradigms that are used to induce affective knowledge. Moreover, it extends the previous research by presenting a very early modulation of perception by socially learned affective knowledge. Importantly, it also offers increased ecological validity of the findings due to the use of naturalistic social exchange in the study design.

## Abstract 2

This research complements previous evidence that experimentally induced socio‐affective knowledge about other individuals, modulates processing of their faces, and shows that negative (but not positive) affect enhances very early face processing (the P1). Importantly, we provide an effective affect induction tool—an interactive social exchange game—which offers increased social ecological validity in experimental settings.

## 1 INTRODUCTION

Face processing is immersed in and augmented by context. For example, naturally occurring faces are perceived from the perspective of an individual's rich socio‐cognitive emotional background. Thus, when processing a face, we use not only the immediate information present within and around it (e.g., body context and emotional expression; Aviezer et al., 2011; Eimer & Holmes, 2007, respectively) but also prior knowledge about the observed individual (for a review of internal and external cues in face processing; see Wieser & Brosch, 2012). Importantly, previously acquired knowledge about other people may be emotionally charged and could influence perceptual processing of their faces regardless of emotional expressions. Indeed, this modulation has been observed in studies in which perceptual face processing of individuals with neutral facial expressions was influenced by experimentally induced and emotionally valenced judgments about them (Bliss‐Moreau et al., 2008; Todorov & Evans, 2007), self‐ or other‐related information (Schwarz et al., 2013; Wieser et al., 2014), affective biographical knowledge (Suess et al., 2015), and other emotional social contexts (Galli et al., 2006; Morel et al., 2012).

By providing participants with descriptions of the behavior of persons in paired photographs, these studies used external explicit sources of information to induce affective knowledge. However, since personal relevance is a significant factor in social cognition (enhancing visual awareness, memory, attention, decision making; e.g., Sui et al., 2015, and modulating face processing; Herbert et al., 2013), acquisition of affective knowledge through personal experience and social interaction may constitute a more robust (and natural) experimental manipulation. In laboratory settings, an interactive social context can be created with the use of various economic games, such as the Prisoner's Dilemma game, the Ultimatum Game, or the Trust Game (Rilling & Sanfey, 2011). In this vein, Singer et al. (2004) utilized an iterated version of the Prisoner's Dilemma game to enable social learning of the moral status of coplayers. They observed that acquired affective knowledge (1) facilitated recognition of the faces used in the study and (2) altered neural activity in the brain structures associated with social cognition and emotions (amygdala, insula, fusiform gyrus, reward‐related areas, and superior temporal sulcus). In the present study, we extended the method used by Singer et al. (2004) to investigate the impact of socially induced affective knowledge on face processing.

### 1.1 The influence of prior social knowledge on early perceptual stages of face processing

Prior knowledge and experiences have been repeatedly shown to modulate very early (even less than 100 ms after stimulus onset) neural responses (Abdel Rahman & Sommer, 2008; Bao et al., 2010; Bar et al., 2006; Chaumon et al., 2008, 2009; Gamond et al., 2011; Pourtois et al., 2008). Specifically in the social domain, familiarity and personal relevance have previously been observed to exert an important influence on visual processing of faces (Ramon & Gobbini, 2018), for example, dramatically facilitating recognition (Natu & O'Toole, 2011) and enhancing early neural processing (e.g., increased P1 amplitudes in Bayer et al., 2021). Crucially, discrimination of facial identity, which requires prior social and semantic knowledge, has been reported to occur very early (even as early as 70 ms after stimulus onset; Nemrodov et al., 2016), especially for familiar (as compared to unfamiliar) faces (Dobs et al., 2019). Also, self‐reference (one of the aspects of personal relevance) has been shown to modulate both early and late brain responses to faces (Wieser et al., 2014). In addition, recent research has provided evidence for very early processing differences between positively and negatively associated faces with neutral expressions (Abdel Rahman & Sommer, 2012; Morel et al., 2012). Thus, overall, previous findings indicate that socio‐affective knowledge (i.e., emotionally charged knowledge about others) about other individuals modulates very early perception of their faces, which may be further facilitated by familiarity and personal relevance.

The reason for this early modulation may lie in the fact that prior knowledge charges otherwise neutral stimuli with salience, which boosts attention allocation and prioritizes perceptual processing of these stimuli (Pourtois et al., 2013). This is advantageous from the evolutionary perspective in the social domain: Early recognition of valence associated with the face of another person may be adaptive in terms of survival (e.g., threatening individuals with aggressive expressions). When searching for the mechanism underlying this modulation, Abdel Rahman and Sommer (2008, 2012) proposed that high‐level semantic knowledge about objects may influence perception through either reentrant activation from higher‐order to sensory structures or through restructuring of the areas devoted to perceptual analysis. In this way higher‐order brain structures may exert an influence on the areas devoted to perception, and prior (social) knowledge may penetrate perceptual processes virtually as early as information becomes available.

### 1.2 The influence of prior social knowledge on attention

Not surprisingly, social knowledge about other individuals has also been repeatedly reported to influence attentional processes, including gaze cueing of attention. Following the gaze of others is a critical adaptive mechanism in the social life of humans, as it allows us to understand which elements of the surrounding environment others pay attention to and that they may have different knowledge than us due to their different visual point of view (Davidson & Clayton, 2016). The impact of socio‐affective knowledge on such attentional mechanisms has frequently been studied using faces as cues that provide information about the location of future targets in attentional cueing tasks (Dalmaso et al., 2020). The most popular paradigm is a variation of the Posner task (Posner, 1980), which offers a well‐established and validated experimental framework with a robust cueing effect (Reppa et al., 2012), also in the social domain (most commonly gaze‐cueing).

Although both perceptual features conveyed by cueing faces (like emotional expressions) and social factors extracted as a result of higher‐level processing (like social status) can influence gaze cueing of attention, the literature is not conclusive about the nature of these effects (Dalmaso et al., 2020). For example, the results of studies examining the link between emotional expressions and attentional cueing effects are inconsistent: some of them show no emotional modulation, whereas others point to facilitated attentional shifts after the presentation of positive or negative emotional faces (for a comprehensive list, see Dalmaso et al., 2020). Moreover, these findings seem to be influenced by many factors, ranging from observers’ individual differences (e.g., gender), through the static/dynamic nature of the stimuli (pictures or short videos), to technical aspects of the experimental designs (time between the cue and the target, frequency of the stimulus' presentation).

In contrast, research on the link between higher‐level social factors (like physical dominance and social status) and gaze cueing of attention produces a more consistent picture: the higher the social status or dominance of the cueing face, the greater the facilitation of attentional shifts (Dalmaso et al., 2012, 2014; Ohlsen et al., 2013). Thus, it is possible that more complex social knowledge, which originates not only from perception of a face but essentially from the interplay between the perceived face and the observer, may be even more robust in terms of guiding the observer's attention than low‐level face characteristics.

### 1.3 Present study

Although social interactions are crucial for the everyday life of humans, relatively few studies have explored their subsequent influence on gaze cueing of attention. Thus, in this study we aimed to investigate the influence of prior socio‐affective knowledge on early processing of faces which guided the observers' attention by providing gaze cues about the location of future targets. For these aims, we utilized an exchange game (the Trust Game; Rilling & Sanfey, 2011) in which participants acquired personal knowledge about their coplayers through interactions. With this, we aimed to create a natural social situation which would provide increased ecological validity. To maintain experimental control, the coplayers were in reality algorithms programmed to elicit neutral, positive, and negative affect towards themselves. Next, we used a version of the spatial gaze‐cueing task (Posner, 1980) in which locations of future targets were cued by the gaze of faces previously associated with socio‐affective knowledge through naturalistic interactions in the game.

To explore the early stages of facial processing, we collected the brain responses of the participants in a gaze‐cueing task with electroencephalography (EEG), which offers excellent temporal resolution. We specifically targeted the P1 and the N170 event‐related potentials (ERPs), both of which are indicators of early face processing. N170 is an early negativity that is traditionally linked to selective processing of faces, but it is debatable whether it is influenced by previous socio‐affective knowledge associated with neutral faces (Abdel Rahman & Sommer, 2012; Galli et al., 2006; Wieser et al., 2014). P1, a positive potential peaking around 100 ms after stimulus onset, has also been proposed to be face sensitive (for a discussion, see Rossion & Jacques, 2011), and a growing body of literature provides evidence for P1 being influenced by emotional and motivational features of stimuli (Bayer et al., 2012; Delplanque et al., 2004; Hammerschmidt et al., 2017; Morel et al., 2009; Pourtois et al., 2004; Rellecke et al., 2012). Moreover, abnormal P1 has been regarded as a marker of deficits in face processing and affect recognition in clinical populations (Earls et al., 2016), thus indicating its relevance for social and emotional contexts, which are crucial in this study. Additionally, both N170 and P1 have been linked to spatial attention (Holmes et al., 2003; Mangun, 1995), and P1 has been shown to reflect attention allocation in gaze‐cueing paradigms (Lassalle & Itier, 2013; Schuller & Rossion, 2001), indexing the gaze‐cueing effect (facilitated spatial attention to the gazed‐at location). To our knowledge, only one ERP study has targeted the effects of prior affective knowledge in an attentional gaze‐cueing task (Suess et al., 2015), but these results were not included in the published article. Here we contribute to and extend the literature by targeting ERPs in a gaze‐cueing task in which neutral faces were previously linked to affective knowledge by means of personal social interactions.

We suspected that personally acquired affective information about an individual is likely to influence early perceptual processing of this individual's face, as measured by ERPs. As the amplitude of P1 is larger for emotional stimuli, and particularly for negative (rather than positive) emotional stimuli (Smith et al., 2003), we expected to observe increased P1 amplitudes in response to pictures of emotionally associated players (and especially of the player who had previously been associated with negative affect) in comparison to pictures of neutral players. As the literature is unclear about the effects of affective knowledge on N170, we did not have a directional hypothesis for this component. Furthermore, we hypothesized a greater cueing effect for the negative player: The induced affective knowledge would influence behavioral responses in the gaze‐cueing attentional task, in such a way that the reaction times to targets congruently cued by the negative player would be the shortest. Although the literature is inconclusive about this effect (some studies show a stronger gaze‐cueing effect for “positive”, e.g., more trustworthy individuals [Süßenbach & Schönbrodt, 2014] and some for “negative” ones, e.g., associated with norm‐violating behaviors [Carraro et al., 2017]), we based our prediction on the negativity bias in attention allocation (Baumeister et al., 2001), which predicts faster recognition of negative stimuli and possible earlier attentional processing.

## 2 METHOD

### 2.1 Sample

Thirty‐four volunteers participated in the study: 25 females and 9 males. The average age of the sample was 23.41 (*SD* = 4.26). Genders did not differ in age, *t*(13.73) = −0.29, *p* = .76. Twenty‐six participants were right‐handed; all were in good health and had normal or corrected‐to‐normal vision. All participants were recruited via flyers on the campus of Jagiellonian University in Krakow and via social media. They were compensated with 25 PLN (equivalent to 6 USD). All participants provided written informed consent; the study was approved by the Research Ethics Committee at the Philosophical Faculty of Jagiellonian University in Kraków, Poland, and was conducted in accordance with the Declaration of Helsinki.

### 2.2 Procedure

The experiment consisted of three parts in the following order: induction procedure (game), manipulation check (questions), and gaze‐cueing task. All computerized procedures were prepared in the Python language (Python Software Foundation). At the end of the experiment, participants were debriefed about the aim of the study and the included deception.

### 2.3 Induction procedure

The exchange game used to induce affective knowledge was an iterative version of the Trust Game (e.g., Rilling & Sanfey, 2011), which has been used in our group previously and is described in detail elsewhere (Senderecka et al., 2020). In short, participants played with three other players with the aim of maximizing their own outcomes. Participants were told that the other players were located in different labs and were subjects in the same study; they interacted via an online game interface and could see each other's photos and exchange short messages. In reality, the two players were algorithms designed to either play fairly, thus maximizing their own and the participant's winnings (see Game structure for details), or unfairly, thus winning more money than the participant. The third player served as a control condition and thus could not be associated with any affect. With this aim, participants were told that there was a technical error and this player could not connect with them; their picture stayed on the screen to ensure balanced exposure time of each of the three players' faces.

#### 2.3.1 Game structure

**FIGURE 1:**
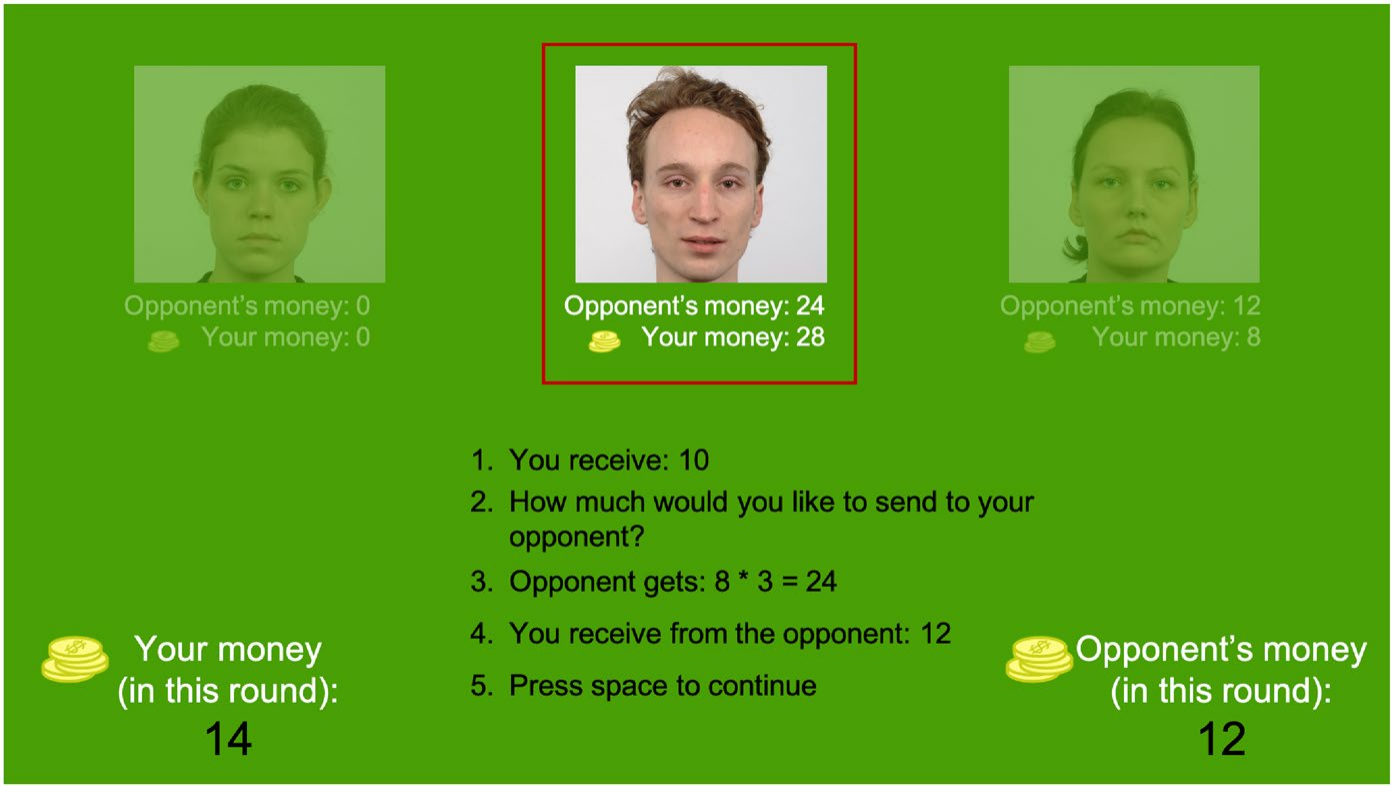
Game display. On the top part of the screen, pictures of all three coplayers are displayed. The player/opponent in the current round is marked with a red frame. In the depicted example participant receives 10 PLN (point 1) and decides to send to the opponent 8 (point 2). The opponent receives this amount tripled (point 3) and “decides” to share half of it with the participant (point 4; in the example the current opponent is the positively associated player). In the bottom right and left corners, the amount of money in the current round for the participant and the opponent are displayed

An exemplary game moment is depicted in Figure 1. In each turn in the game, one player (the investor) received 10 PLN and had to send some or all of this to their current coplayer (the trustee). The amount was tripled in the transmission. The trustee could (but did not have to) return some or all of the received money to the investor. Since the game was iterated, the rational choice for the trustee was to send back half of the received amount in order to play fairly and to increase the chance that the investor would be willing to send the maximum amount (10 PLN) in the next rounds (sending only a little money back may make the investor less likely to send large amounts in the future). Hence, to ensure maximum winnings for both players, the investor should send all the money (10 PLN) to the trustee, who receives the tripled amount (30 PLN) and should send half of it back to the investor, thus leaving both players with 15 PLN each. On the other hand, the investor could send 1 PLN to the trustee, which would leave the players with 9 and 3 PLN, respectively (since the trustee is not likely to send anything back). Although this strategy ensures that the investor wins against the trustee, it is not the optimal choice in a game with four players as another dyad might choose the maximizing strategy that results in higher winnings (15 vs. 9 PLN). Therefore, the positive player always sent back half or slightly more of the received money, and the negative player either kept the whole amount or sent a small portion back. The whole game consisted of six subgames played in a randomized order between the four players: the participant, the positive player (POS), the negative player (NEG), and the neutral player (NEU). Each subgame was divided into four blocks of three turns. The game was preceded by a training block with a computer that displayed a simply sketched face as the dummy co‐player's picture. Participants were informed they would receive 50, 25, or 10 PLN according to the place taken in the game (1st, 2nd, and 3rd). In reality, all participants were placed second (and NEG came first).

#### 2.3.2 Deception mechanisms

To increase the believability of the players, a number of scripted elements were administered. First, participants were asked to pose for pictures (neutral expression, straight, and averted gaze) to justify the photos of other players displayed in the game and the gaze‐cueing task. The photos were then “sent” to a study assistant who made sure they all looked similar. Before the game, the participants flipped a coin to establish their role (investor/trustee), which, unknown to them, always resulted in them being investors. Furthermore, at the beginning of the game each player sent a welcome message to the others. POS sent a cheerful message with a smiley emoticon, a wish of good luck, and an exclamation mark; NEU sent a simple welcome; NEG sent a cold and restrained “hi” followed by a period. In the middle of the game, the participants could send a message to their coplayers; in return, they received short, impersonal but positive (“after all, not a bad game, is it?”) or negative (“I’ve played more thrilling games before...”) messages from the POS and NEG players, respectively. The messages contained a typo to further aid the belief in a fellow human player. Finally, the technical error that occurred for NEU was accompanied by a fake call to a researcher at the other study site in which the error was apologized for and it was mentioned that the other lab experienced occasional internet connection problems.

#### 2.3.3 Stimuli

The pictures of faces used in the game were selected from Radboud Faces Database (Langner et al., 2010, www.rafd.nl). Twelve pictures of young White people (six men and six women) facing the camera with a straight gaze and neutral expressions were used. For each participant, three faces were chosen pseudo‐randomly (controlling for the frequencies of photos in the study as well as the gender of the faces in the photos), one of which was a different gender than the other two. The same set of three faces never occurred more than once across participants, that is, a set of three of the same identities ascribed to the three conditions (e.g., photo number 1 as a POS, 2 as a NEG, and 3 as a NEU) was never used twice in the study (the photos were reused across participants but as different players: POS, NEG, NEU).

### 2.4 Manipulation check

After the game, participants were shown all twelve pictures used in the study (three known to them from the game and nine unknown) and were instructed to report whether they recognized the person depicted as one of the game players. This face recognition task was designed to ensure that participants paid attention to the faces during the game, which was a crucial step in associating a face with an affect. Two participants, once each, wrongly reported not recognizing a person in a picture, that is, they indicated that the person was not one of the three players from the game. All the analyses described in the results section were conducted with and without exclusion of these two participants and yielded similar results. Thus, they were not excluded from the study sample.

Following the face recognition task, participants answered questions displayed on a computer screen and gave their responses on a scale ranging from 1 (not at all) to 5 (very much). The first two, “how much did the game engage you emotionally?” and “how much did you like the game?”, were control questions (intended to reveal a potential overall lack of engagement or negative experiences associated with the game) and were not subjected to subsequent analysis (the means and standard deviations were 3.2 (1.1) and 3.4 (1.3), respectively). The question “how much did you come to like this player?” was asked separately for each of the three faces (photos simultaneously displayed with the questions) and was designed as a manipulation check for the affective knowledge induction.

### 2.5 Gaze‐cueing task

Participants were prepared for the EEG recording and seated approximately 60 cm from a 23‐inch color monitor in a dimly lit and electrically shielded room. They performed a variation of the Posner task (Posner, 1980), in which gaze‐cueing was administered with pictures of faces known to each participant from the previous game.

**FIGURE 2:**
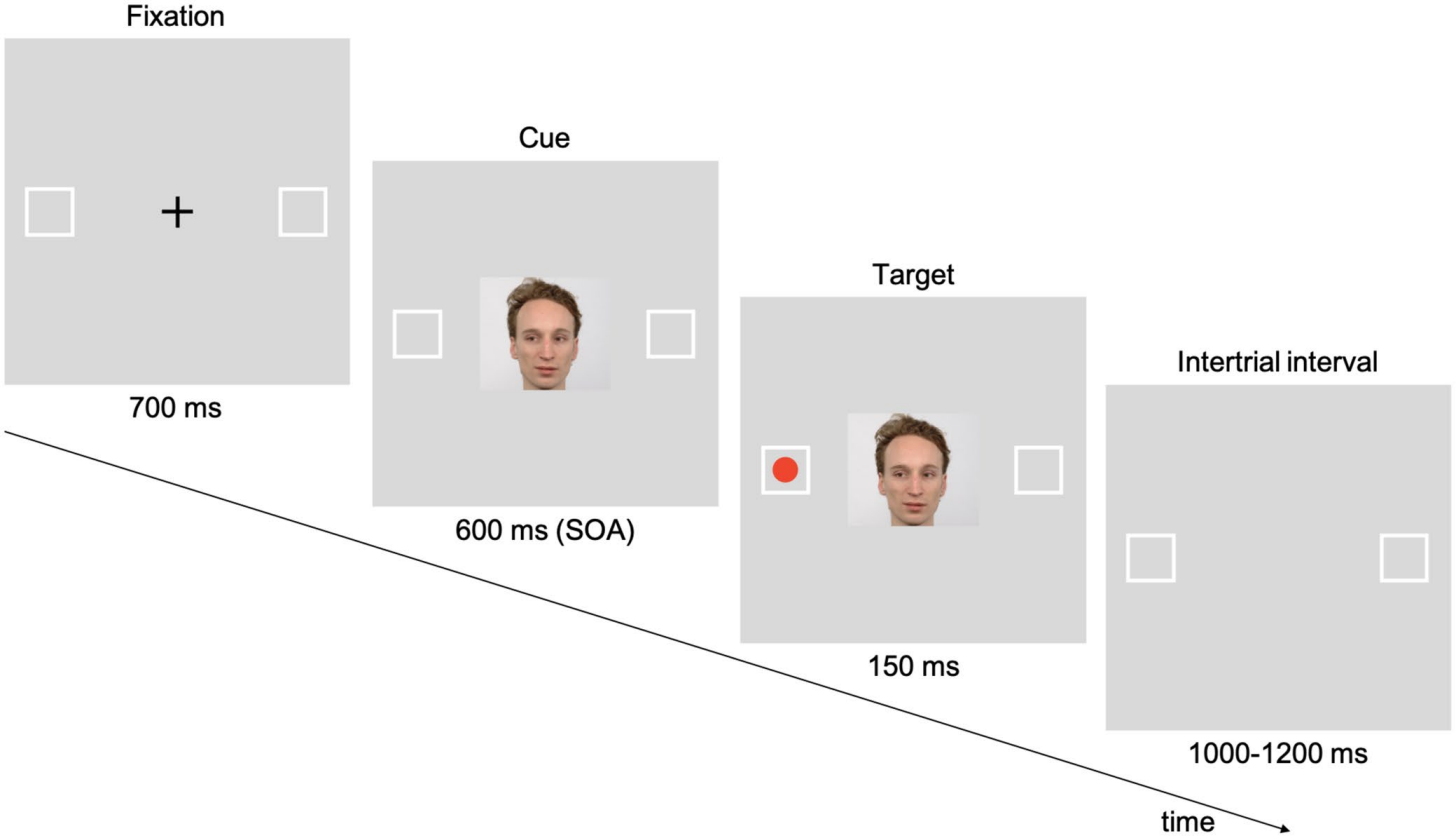
Experimental design of the gaze cueing task. The presented example shows a valid (congruent) trial, in which after 700 ms of the fixation point display, a picture of a face looking left appears, followed by a 150‐ms presentation of the target in the left square (previously cued location). Another type of a valid trial would include a face looking right followed by the target appearing in the right square. Each trial finishes with an intertrial interval of 1,000–1,200 ms

In each trial, a 1 × 1 cm fixation cross was first displayed in the middle of the screen, with two empty squares (3.5 × 3.5 cm) positioned 19 cm to the left and to the right. Participants were instructed to fixate their gaze on the cross. After 700 ms, a cue in the form of one of the faces was presented. All of the faces had a neutral emotional expression, and the gaze looked straight forward, right or left. The orders of both the face identity (POS, NEG, and NEU) and the gaze direction were randomized, and their frequency was equally distributed. After 600 ms (stimulus onset asynchrony) a red dot (diameter 0.8 cm) appeared in one of the squares for 150 ms (target). When the eye gaze of the presented face was followed by a target matching the gazed‐at location, it constituted a congruent trial. In incongruent trials the gaze was directed at the location that the following target did not appear in; in uncued trials the gaze of the presented face was straight (no location cueing). The faces' identities (POS, NEG, and NEU) constituted conditions; the faces of players associated with positive, negative, and neutral affect marked trials in the POS, NEG, and NEU conditions, respectively. The participants were asked to detect the target and report its position as quickly as possible by pressing left or right Ctrl key, respectively, with their index fingers. As soon as the response was given or after 500 ms and no response (invalid trial), the cue, the target, and the fixation cross disappeared, and the intertrial interval was introduced for 1,000–1,200 ms. An overview of a trial is presented in Figure 2. In total, 600 trials were administered in six blocks, following one block of training during which simply sketched dummy faces were used as cues. Participants' brain activity and response times were measured throughout the task.

### 2.6 EEG recording and signal processing

The continuous scalp electroencephalogram (EEG) was recorded from 32 silver/silver‐chloride (Ag/AgCl) active electrodes (with pre‐amplifiers) using the BioSemi Active‐Two system. The electrodes were secured in an elastic cap (Electro Cap) according to the extended 10–20 international electrode placement system. The signal was continuously recorded at 256 Hz and referenced online to the CMS‐DRL ground, which drives the average potential across all electrodes as close as possible to amplifier zero. The electrode offsets were kept within the range of ±20 µV. The horizontal and vertical electro‐oculograms (EOGs) were monitored using four additional electrodes placed above and below the left eye and in the external canthi of both eyes. All channels were re‐referenced offline to the average of all the electrodes. The recordings were filtered offline with a high‐pass filter of 0.05 Hz (slope 24 dB/oct) and a low‐pass filter of 25 Hz (slope 12 dB/oct); they were then manually inspected to remove nonstationary artifacts (such as skin potentials or artifacts due to head movements). Ocular and other stationary artifacts were removed with the independent component analysis algorithm (ICA) using Brain Vision Analyzer 2 (Brain Products, Munich, Germany). After removal of ocular artifacts, a semi‐automatic artifact‐rejection procedure was conducted with a minimum and maximum allowed amplitude of −65 and 65 µV, respectively. Continuous data were segmented into epochs ranging from −100 ms before to 600 ms after the cue onset. All segments were aligned to a 100‐ms precue baseline. Across participants, an average of 199 artifact‐free trials was obtained in each condition (POS: *SD* = 0.9, NEG: *SD* = 0.39, NEU: *SD* = 0.92). Finally, epochs were averaged per subject and condition. The time window and the region of interest for P1 and N170 in response to cues were chosen based on prior research (Luck, 2005; Nemrodov et al., 2016). The time windows of interest for P1 and N170 were respectively 90–140 and 140–190 ms after stimulus onset. The averages were calculated from electrodes PO3, O1, Oz, O2, and PO4 for P1 and from electrodes P8, PO4, P7, and PO3 for N170.

### 2.7 Data analysis

All data analyses were performed using R ver. 3.6.1 (R Core Team, 2019). The significance level for all the tests was set to 0.05. We utilized multiple regression analyses with mixed effects through the lmerTest package ver. 3.1‐0 (Kuznetsova et al., 2017). Based on the experimental design (condition nested in subjects) and following Akaike's Information Criterion (Akaike, 1998), the random intercepts for subjects were added to the intercept‐only model. Assumptions for multiple regression (normality, linearity, multicollinearity, and homoscedasticity) were checked. Marginal and conditional *R* ^2^ were calculated as measures of goodness of fit for the mixed models (Nakagawa & Schielzeth, 2013), in which marginal *R* ^2^ (*R* ^2^m) reflects variance explained by fixed factors, and conditional *R* ^2^ (*R* ^2^c) reflects the variance explained by the entire model. The *p*‐values were computed via Wald‐statistics through the lmerTest package and were then corrected for multiple comparisons with the Bonferroni method. Treatment contrasts were utilized in all models. To estimate the main effect of multilevel categorical predictors (condition and congruency), we used analysis of variance (ANOVA) with Satterthwaite approximation for degrees of freedom and type II sums of squares on the regression models (Kuznetsova et al., 2017). Since there is no established way of calculating standardized effect sizes for linear mixed models, see the unstandardized slope estimates for the essential effect size statistics (Pek & Flora, 2018).

Participants' answers to questions about their emotional attitude towards players were subjected to nonparametric Friedman and Wilcoxon tests (as the answers given on a 5‐point scale did not follow a normal distribution).

To explore the influence of condition on P1, we built a regression model with condition (POS, NEG, and NEU) as the main predictor. For the analysis of N170, to comply with the convention and to address the commonly observed effects of right‐lateralization of this component (Luck, 2005), in addition to condition, we included hemisphere (right, left; electrodes P8 and PO4, P7, and PO3, respectively) as a predictor of no interest to the hypothesis testing. For the analysis of condition and congruency (congruent, incongruent, no‐cue) effects on reaction times, both were included in the model as predictors with an interaction term.

Additionally, we conducted exploratory analyses of the influence of subjective ratings as a predictor (instead of predefined conditions), which allow for more refined conceptualisation of the intersubjective variability. These analyses can be found in the Supplementary Material to this article.

The script with all the analyses presented here can be viewed in the accompanying file found online (https://osf.io/zvgkw/).

## 3 RESULTS

### 3.1 Manipulation check

The results of the emotion induction check (as revealed by the Friedman test) indicated significant differences between participants' emotional attitudes towards the negative, positive, and neutral players: *χ* ^2^ (2) = 50.46, *p* < .001, *W* = 0.74. Paired comparisons between the likeability ratings using the Wilcoxon test (and Bonferonni multiple testing correction) showed that all the differences were significant beyond the .001 level. These results suggest that the manipulation was successful: participants liked the positively associated players (*M* = 3.94, *SD* = 0.65) more than the neutral players (*M* = 2.97, *SD* = 0.39) and the neutral players more than the negatively associated players (*M* = 2.00, *SD* = 0.78).

### 3.2 Brain responses

The means and standard deviations for P1 and N170 in each experimental condition (and hemisphere for N170) are summarized in Table 1.

**TABLE 1:** Mean amplitude and standard deviation of P1 and N170 for all conditions

Mean amplitude (μV) in experimental condition	POS	NEG	NEU
*P1 component*			
	4.24 (2.93)	4.43 (2.76)	4.07 (2.75)
*N170 component*			
Global	0.29 (2.43)	0.22 (2.39)	0.13 (2.29)
Left	0.50 (2.17)	0.51 (2.10)	0.34 (2.03)
Right	0.08 (2.67)	−0.07 (2.65)	−0.09 (2.53)

#### 3.2.1 The P1 component

**FIGURE 3:**
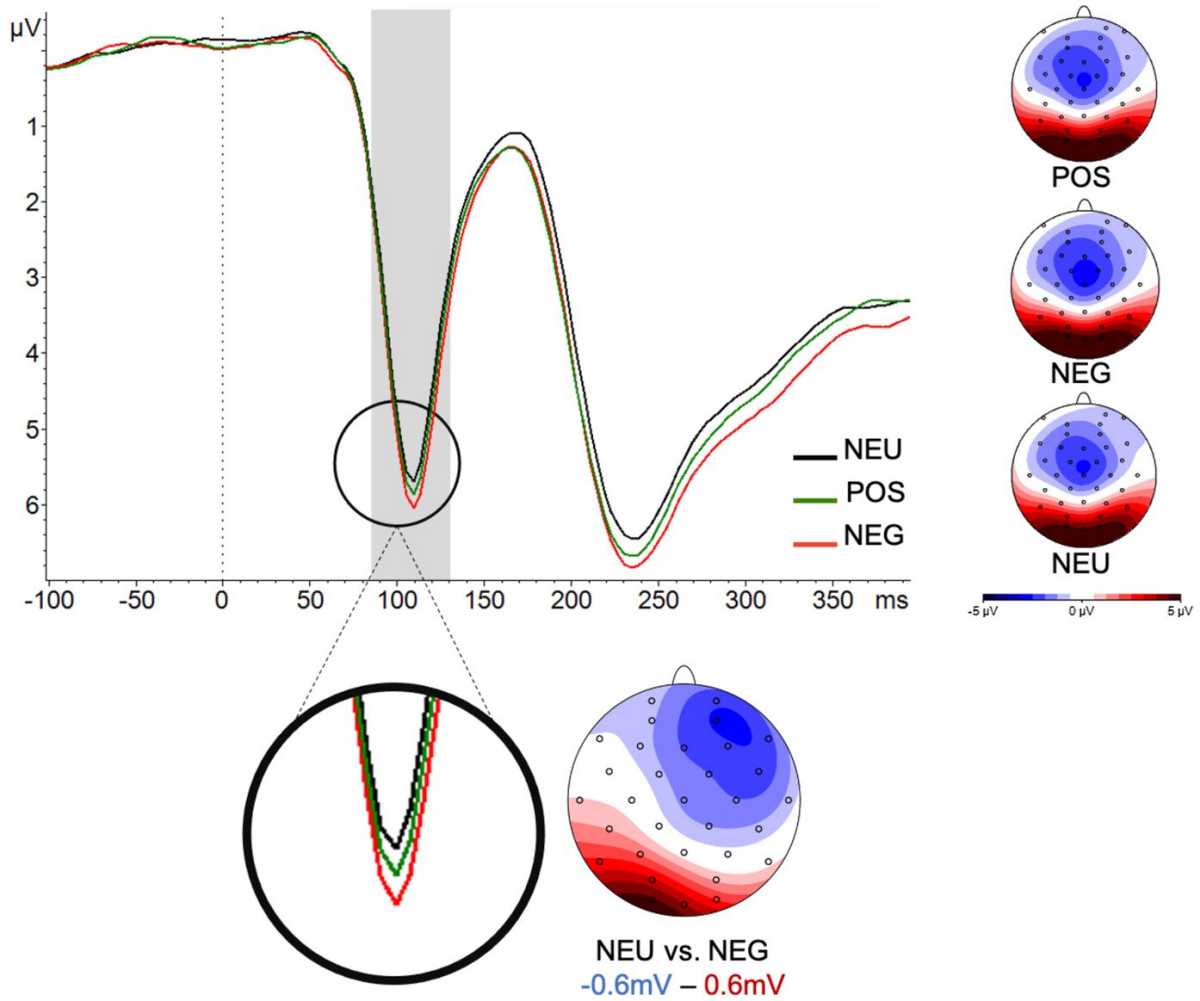
Average amplitudes of the P1 component in response to cue. The averages are plotted for all the electrodes in the region of interest (PO3, O1, Oz, O2, and PO4). Please note that according to the convention, negative values are plotted upwards. The gray shading marks the time window which was used for the analysis (90–140 ms after stimulus onset). The maps show topographies of P1 for each condition (on the right side) and for the difference between the neutral and the negative condition

The amplitudes of the P1 component were time‐locked to the onset of the face cue in the gaze‐cueing task (shown in Figure 3) and were statistically significantly influenced by the condition, *F*(2,68) = 5.77, *p* = .005. Pairwise comparisons revealed that this effect was driven by the difference between NEG and NEU, *est*. = 0.37, 95% *CI* = [0.15–0.58], *t* = 3.4, *p_corr_* = .003. Other comparisons did not reach statistical significance: NEG versus POS, *est*. = −0.19, 95% *CI* = [−0.04 to 0.02], *t* = −1.78, *p_corr_* = .24; NEU versus POS, *est*. = 0.18, 95% *CI* = [−0.04 to 0.39], *t* = 1.62, *p_corr_* = .33. We also tested whether a random intercept for items (pictures of faces used in the study) improved the model, but its addition to a null model (*AIC* = 501.84) did not improve the fit (*AIC* = 503.83, *p* = .98).

An additional exploratory analysis with the P1 as the dependent variable and subjective ratings of participants' emotional attitude towards the players as the predictor yielded a main effect of the ratings, *F*(1,68.17) = 5.83, *p* = .02 (please note that this is an exploratory analysis and the p value should be treated with caution) with a negative estimate of −0.12 (the higher (more positive) the ratings, the smaller the P1 amplitude).

#### 3.2.2 The N170 component

N170 showed a main effect of lateralization, *F*(1,170) = 9.45, *p* = .003 but no main effect of condition, *F*(2,170) = 0.37, *p* = .69. Thus, N170 was more pronounced on the right side (*M* = −0.03 μV, *SD* = 2.6) than on the left side (*M* = 0.45 μV, *SD* = 2.08), but the condition did not modulate its amplitude.

### 3.3 Reaction times

The reaction times were preprocessed in three steps: (1) first we rejected invalid (no response) and incorrect (location of the target reported incorrectly) trials, which accounted for 0.23% and 1.81% of all trials, respectively; (2) then we rejected all trials in which the reaction time was faster than 150 ms (2.70% of trials); and (3) finally, we rejected trials which were larger or smaller than 2 *SD* of each participant's mean reaction time (5.16%). In total, these steps resulted in rejection of 9.99% of all trials.

**FIGURE 4:**
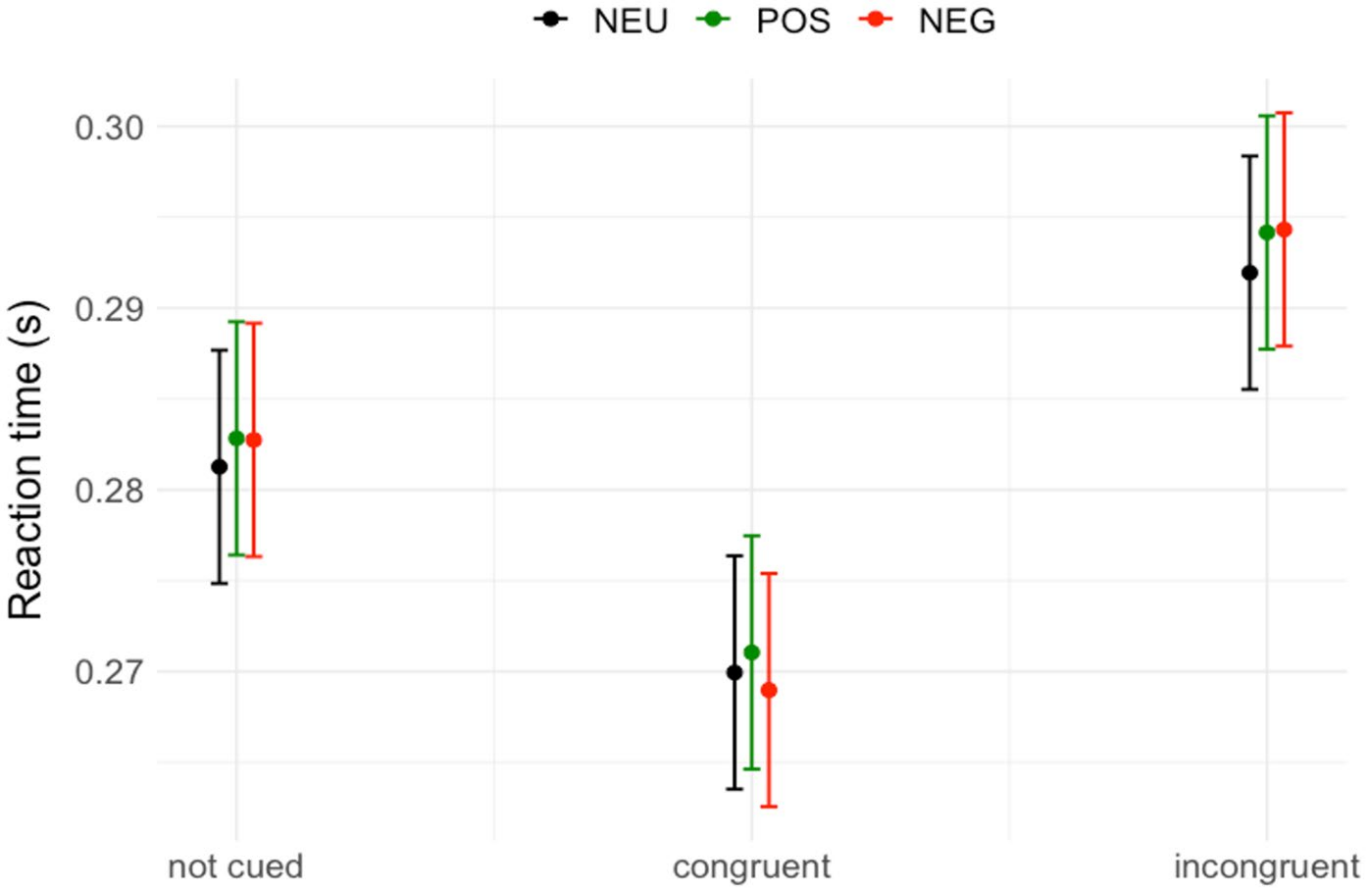
Predicted reaction times grouped by congruency and condition. Error bars represent 95% confidence intervals

The reaction times in the remaining trials were analyzed in a model with congruency (congruent, incongruent, and uncued) and condition (NEU, POS, and NEG) as predictors. ANOVA on the model yielded the main effect of congruency, *F*(2,272) = 159.03, *p* < .001, but did not yield statistically significant effects of condition, *F*(2,272) = 0.77, *p* = .46, or interaction of the two, *F*(4,272) = 0.31, *p* = .87. The reaction times were the slowest in the incongruent condition (*M* = 0.29, *SD* = 0.02), faster in the uncued condition (*M* = 0.28, *SD* = 0.02), and fastest in the congruent condition (*M* = 0.27, *SD* = 0.02). All means and standard deviations across conditions and congruency are presented in Table 2. All pairwise comparisons were statistically significant: uncued versus congruent, *est*. = −0.01, 95% *CI* = [−0.02 to −0.01], *t* = −6.03, *p_corr_* < .001; uncued versus incongruent, *est*. = 0.01, 95% *CI* = [0.01–0.02], *t* = 5.08, *p_corr_* < .001; congruent versus incongruent, *est*. = 0.03, 95% *CI* = [0.02–0.03], *t* = 11.11, *p_corr_* < .001. Figure 4 depicts the predicted values in the model. Altogether, these results suggest that while the effect of congruency on gaze‐cued attentional shift was strong, the condition (affect towards the face) did not modulate the behavioral responses in the gaze‐cueing task.

**TABLE 2:** Means and standard deviations of reaction times across conditions and congruency in the gaze‐cueing task

Mean reaction times and *SD* [s]	POS	NEG	NEU	
Not cued	0.283 (0.015)	0.283 (0.019)	0.281 (0.018)	0.282 (0.017)
Congruent	0.271 (0.019)	0.269 (0.017)	0.270 (0.019)	0.270 (0.018)
Incongruent	0.294 (0.021)	0.294 (0.023)	0.292 (0.023)	0.293 (0.022)
	0.283 (0.021)	0.282 (0.022)	0.281 (0.022)	

Note

Due to small differences, the reported numbers are rounded to three decimal places.

## 4 DISCUSSION

The aim of this study was to test whether affective knowledge induced through naturalistic social learning in an interactive exchange game influences early stages of face processing and attentional shifts in a subsequent gaze‐cueing task. To this goal, we used an iterative version of the Trust Game in which a cooperative player was associated with positive affect, a defecting player with negative affect, and an absent player with neutral affect. The photographs of the players were then used in a variation of the Posner task in which the locations of the future targets were cued by the gaze of the players. The game was successful in inducing valenced socio‐affective knowledge towards positive and negative players, as expressed by self‐reported ratings. We also observed enhanced early neural activity in response to the photograph of the negative player (expressed as larger amplitude of the P1 component), suggesting that affective knowledge about an individual modulates early stages of their face processing. However, this modulation was insufficient to cause behavioral differences in the gaze‐cueing task. Moreover, no such effect was found for N170.

### 4.1 Evaluation of the affective knowledge induction procedure

Self‐reported ratings after the game confirmed that it was successful in eliciting emotionally valenced knowledge about the players. Therefore, our study replicates and extends previous research that utilized similar socially meaningful interactive contexts (Singer et al., 2004) or explicit socio‐affective information (Bliss‐Moreau et al., 2008; Galli et al., 2006; Morel et al., 2012; Suess et al., 2015; Todorov & Evans, 2007) to induce emotional knowledge about faces with neutral expressions. Specifically, we observed that the cooperative players were rated as more liked than the neutral ones and the latter as more liked than the negative players. Importantly, the difference in these ratings cannot be explained by the facial expressions of the players (as all photographs depicted neutral faces and were randomly assigned across participants) or by random effects of the photographs, as reflected by the lack of improvement of the model's fit upon the addition of a random term for items. Thus, our results indicate that the social learning during the naturalistic exchange game elicited emotional associations with the photos of the players. This is an important finding as it confirms that affective knowledge about individuals can be learned rapidly in a controlled and relatively easy‐to‐administer interactive social scenario, like an exchange game.

### 4.2 Neural responses to emotionally associated faces

On the neural level, we observed that P1 (an early perceptual component) showed an increased amplitude for the negative players. This finding is consistent with literature that shows modulations of early perception by emotionally valenced knowledge (Abdel Rahman & Sommer, 2012; Galli et al., 2006; Morel et al., 2012; Suess et al., 2015). Moreover, it provides a strong argument that face perception is not merely a passive sensory phenomenon but rather an active process penetrated by prior knowledge (e.g., Abdel Rahman & Sommer, 2008; Dobs et al., 2019). Since we did not observe larger P1 amplitude to positive than to neutral players, our findings are also in line with research indicating a general negativity bias in humans (Baumeister et al., 2001): specifically greater saliency of negative than positive or neutral social stimuli (Abdel Rahman, 2011; Pecchinenda et al., 2008; Putman et al., 2006; Suess et al., 2015 but cf. Singer et al., 2004).

It is worth noting that larger P1 responses to negatively associated faces could be driven by memory‐induced distortion of perception that causes the observed face to seem more negative in its expressions. Suess et al. (2015) observed that neutral faces associated with negative biographical information produced more negative expression ratings and enhanced amplitudes of early posterior negativity (EPN, elicited around 200–250 ms after stimulus onset) (see also Abdel Rahman, 2011). They interpreted these results in terms of early perceptual effects of affective knowledge. Crucially, such a pattern of results was not observed for neutral faces associated with positive knowledge, despite being rated as significantly more likeable than faces from the two other conditions, similarly as in our study. Although our results do not provide direct evidence that the observed neural differences were caused by seemingly more negative expressions (we did not collect ratings of emotional expression), it is highly probable that the emotional modulation of the P1 amplitude was driven by a similar mechanism, thus reflecting a negativity bias in the early stages of perceptual processing.

In addition, to view our results in the context of interindividual differences, we built an exploratory model with subjective ratings of the coplayers' likability instead of the predefined condition (POS, NEG, and NEU). This approach allowed to better reflect the variability between subjects and the qualitative value of the stimuli. The analysis revealed that lower ratings were linked to larger P1 amplitudes—a result which parallels our planned analyses (see Supplementary Material for details). Together, the planned and exploratory analyses provide strong evidence for the enhanced early brain responses to negatively associated neutral faces.

As to the N170 component, we did not observe a modulation of its amplitude by affective knowledge. Although a recent meta‐analysis confirmed that the N170 amplitude is influenced by emotional expressions (for a review, see Hinojosa et al., 2015), this effect may be due to structural processing of the face (in contrast to affective knowledge). Importantly, other studies manipulating self/other‐reference and biographical knowledge also reported no modulation of N170 (Abdel Rahman & Sommer, 2012; Wieser et al., 2014 but cf. Galli et al., 2006). However, since the influence of top‐down socio‐affective knowledge on the N170 amplitude is still debated, we cannot rule out that our manipulation was too weak to reveal this effect. Certainly, more studies are needed to form conclusions.

### 4.3 No effects of the induced affective knowledge on behavior

We hypothesized that we would observe shorter reaction times following congruent cues consisting of the pictures of the negative players. Contrary to our predictions (and some earlier studies; Carraro et al., 2017), the induced affective knowledge did not influence the cuing effect in the gaze‐cuing task. Our reasoning was based on the known effects of the negativity bias in attention allocation (Baumeister et al., 2001), indicating that negative stimuli are attended to faster, which accelerates processing and allows faster subsequent motor reactions.

When searching for the reason for the lack of this effect in our data, it should be noted that the task required detecting and reporting locations of cued targets. Thus, the processing of facial features other than gaze direction (i.e., goal‐related cue) was not necessary for successful completion of the task and could be suppressed at later stages of face/emotion processing (at least later than those indexed by P1). Therefore, the affective knowledge induced by social interactions could be unable to modulate behavioral responses to target stimuli. In a broader perspective, the interplay between perceptual, emotional, and goal‐related processing over time in the gaze‐cuing task certainly poses an interesting research question which should be further addressed in future studies.

It is also worth mentioning that some studies found effects of social information on reaction times only in short stimulus‐onset asynchrony (e.g., Dalmaso et al., 2014), which suggests that such effects may decay with time. Hence, another reason for the lack of behavioral effects in our study may lie in the relatively long (600 ms) time interval between the cue and the target used here (this was done to avoid overlapping anticipatory processes of the upcoming performance in the EEG signal).

Finally, although we did not observe behavioral effects in our study, we cannot rule out that the induced affective knowledge could modulate behavior in ways other than increasing or decreasing reaction times in a gaze‐cued attentional task (as hypothesized here). Given that an experimental induction of affective knowledge has been successful in this and previous studies, future research could use similar procedures and subsequently investigate a variety of possible behavioral outcomes in different tasks (e.g., Senderecka et al., 2020). Lastly, it is worth noting that other studies have also previously reported modulations of emotional information on the neural level with no behavioral effects (for a similar design, see Fichtenholtz et al., 2007). Since behavioral responses and brain activity are to some extent dissociable, the effects observed on the neural level certainly do not have to be echoed in behaviorally observed differences (Gratton et al., 2018).

### 4.4 Ecological validity and limitations

By introducing a meaningful social and emotional context, our study improved in terms of ecological validity by approximating a truly interactive environment rather than the mere observation of others. This “second‐person” approach to social neuroscience has gained growing attention over the years as a relevant route to investigation of human social cognition (Schilbach et al., 2013). Although following this promising line was an important aim of this study, we realize that a few limitations can be identified in our design.

First of all, one could argue that economic games are not common or important in daily life, and hence they create an artificial setting. However, an exchange in such games can be regarded as a structured example of social reciprocation and cooperation, both of which are crucial for successful social interactions (Singer et al., 2004).

Furthermore, interaction over an online interface may be lacking in terms of the limited information about the partners (no information from facial expression changes, body posture, movements, prosody) and the lack of physical proximity (“same‐room‐ness”). Although this may be considered a limitation of the ecological validity of our design, digitalization and the increased use of computerized means of communication nowadays probably make such interfaces less artificial than they might have been a few decades ago. Crucially, it also allows better experimental control in studies.

Another limitation of our design is the use of static stimuli (photographs of faces) instead of dynamic ones (Dziobek, 2012), but this was a deliberate decision that was taken to increase the experimental control. Nevertheless, since natural social stimuli are dynamic in nature (Schilbach et al., 2013) and natural familiarization of faces includes variability in viewing conditions (and lesser dependence on image‐based recognition; Ramon & Gobbini, 2018), it would be beneficial to include dynamic representations of game partners in our study design.

Finally, it should be noted that the likability ratings in this study were administered before the attentional task, which does not offer evidence that the social knowledge was maintained in memory throughout the entire length of the task. Future studies should address this limitation by administering the manipulation check after the task (like in Dalmaso et al., 2014 and Carraro et al., 2017). Altogether, though our design is not free of limitations, it significantly increases the ecological validity by introducing a meaningful and interactive social context for induction of personal affective knowledge.

## 5 CONCLUSIONS

In this study, using naturalistic social learning in an interactive exchange game, affective knowledge about the coplayers was induced in the participants. Subsequently, this socio‐affective knowledge (specifically about negative players) influenced very early brain responses to pictures of the coplayers displaying neutral facial expressions. Modulation of such early neural processing by prior knowledge suggests that emotionally charged interactions increase the saliency of the interaction partners' faces. Importantly, the design of the game employed in this study allowed for a naturalistic social setting, which increased ecological validity of the results.

## AUTHOR CONTRIBUTIONS

**Magdalena Matyjek:** Conceptualization; Data curation; Formal analysis; Funding acquisition; Investigation; Methodology; Validation; Visualization; Writing‐original draft; Writing‐review & editing. **Bartłomiej Kroczek:** Data curation; Software. **Magdalena Senderecka:** Conceptualization; Data curation; Formal analysis; Funding acquisition; Investigation; Methodology; Project administration; Resources; Software; Supervision; Validation; Visualization; Writing‐original draft; Writing‐review & editing.

## Supporting information

Supplementary Material
