# Supplementary material for: Socially induced negative affective knowledge modulates early face perception but not gaze cueing of attention
Source: Psychophysiology. 2021 Jun 10;58(9):e13876. doi: 10.1111/psyp.13876 (PMC8459251; doi:10.1111/psyp.13876)
Supplement: Supplementary file 1 — Supplementary Material [file PSYP-58-e13876-s001.docx]

Supplementary material to:

**Socially Induced Negative Affective Knowledge Modulates Early Face Perception but not Gaze Cueing of Attention**

Exploratory analysis of the subjective ratings of the coplayers’ likability as a predictor of the P1 amplitude.

**Model:**

lmerTest::lmer(formula = P1 ~ rating + (1 | ID), data = data,

REML = F)

**Type II Analysis of Variance Table with Satterthwaite's method on the model:**

|  | Sum Sq | Mean Sq | NumDF | DenDF | F value | Pr(>F) |
| --- | --- | --- | --- | --- | --- | --- |
| rating | 1.2449 | 1.2449 | 1 | 68.172 | 5.8337 | 0.01841 |
|  |  |  |  |  |  |  |

**Model summary:**

|  | **P1** | | |
| --- | --- | --- | --- |
| *Predictors* | *Estimates* | *CI* | *p* |
| (Intercept) | 4.60 | 3.63 – 5.56 | **<0.001** |
| rating | -0.12 | -0.21 – -0.02 | **0.016** |
| **Random Effects** | | | |
| σ^2^ | 0.21 | | |
| τ_00_ _ID_ | 7.46 | | |
| ICC | 0.97 | | |
| N _ID_ | 34 | | |
| Observations | 102 | | |
| Marginal R^2^ / Conditional R^2^ | 0.002 / 0.972 | | |
